# Novel peanut-specific human IgE monoclonal antibodies enable screens for inhibitors of the effector phase in food allergy

**DOI:** 10.3389/fimmu.2022.974374

**Published:** 2022-09-29

**Authors:** Jada Suber, Yugen Zhang, Ping Ye, Rishu Guo, A. Wesley Burks, Michael D. Kulis, Scott A. Smith, Onyinye I. Iweala

**Affiliations:** ^1^ Department of Microbiology and Immunology, University of North Carolina at Chapel Hill (UNC), Chapel Hill, NC, United States; ^2^ Department of Medicine, Thurston Arthritis Research Center, Division of Rheumatology, Allergy, and Immunology, University of North Carolina at Chapel Hill (UNC), Chapel Hill, NC, United States; ^3^ Department of Pediatrics, Division of Allergy and Immunology, University of North Carolina Food Allergy Initiative, University of North Carolina School of Medicine, Chapel Hill, NC, United States; ^4^ Department of Medicine, and Department of Pathology, Microbiology and Immunology, Vanderbilt University Medical Center, Vanderbilt University, Nashville, TN, United States

**Keywords:** peanut allergy, human IgE, mast cell, CD300a, Siglec-8

## Abstract

**Background:**

10% of US residents have food allergies, including 2% with peanut allergy. Mast cell mediators released during the allergy effector phase drive allergic reactions. Therefore, targeting sensitized mast cells may prevent food allergy symptoms.

**Objective:**

We used novel, human, allergen-specific, IgE monoclonal antibodies (mAbs) created using human hybridoma techniques to design an *in vitro* system to evaluate potential therapeutics targeting sensitized effector cells.

**Methods:**

Two human IgE mAbs specific for peanut, generated through human hybridoma techniques, were used to sensitize rat basophilic leukemia (RBL) SX-38 cells expressing the human IgE receptor (FcϵRI). Beta-hexosaminidase release (a marker of degranulation), cytokine production, and phosphorylation of signal transduction proteins downstream of FcϵRI were measured after stimulation with peanut. Degranulation was also measured after engaging inhibitory receptors CD300a and Siglec-8.

**Results:**

Peanut-specific human IgE mAbs bound FcϵRI, triggering degranulation after stimulation with peanut in RBL SX-38 cells. Sensitized RBL SX-38 cells stimulated with peanut increased levels of phosphorylated SYK and ERK, signal transduction proteins downstream of FcϵRI. Engaging inhibitory cell surface receptors CD300a or Siglec-8 blunted peanut-specific activation.

**Conclusion:**

Allergen-specific human IgE mAbs, expressed from human hybridomas and specific for a clinically relevant food allergen, passively sensitize allergy effector cells central to the *in vitro* models of the effector phase of food allergy. Peanut reproducibly activates and induces degranulation of RBL SX-38 cells sensitized with peanut-specific human IgE mAbs. This system provides a unique screening tool to assess the efficacy of therapeutics that target allergy effector cells and inhibit food allergen-induced effector cell activation.

## Introduction

Food allergies affect 10% of the US population, including 2%, or over 6 million people, with peanut allergies (1, 2). There is no cure for peanut allergy, and individuals affected are at greater risk of anaphylaxis compared to those with other allergies (3).

Allergic reactions are driven, in large part, by mast cells (MCs) during the allergy effector phase (4). During this phase, allergenic antigens crosslink allergen-specific immunoglobulin (Ig)E bound to the IgE receptor, FcϵRI, on MCs, activating these cells. When activated, MCs drive allergic symptoms by degranulating and releasing pre-formed mediators. These mediators, stored in MC cytoplasmic granules, include proteases, vasoactive amines like histamine, and the cytokine tumor necrosis factor (TNF)-alpha. MCs also synthesize *de novo* lipid mediators and additional cytokines to maintain allergic symptoms (4). Thus, targeting MC activity during the allergy effector phase may prove useful for developing new therapeutics to treat food allergy.

MC inhibitory receptors, like CD300a and sialic acid-binding immunoglobulin-like lectin (Siglec)-8 mitigate allergic inflammation and MC degranulation in passive cutaneous anaphylaxis models (5, 6) and a murine allergic peritonitis model (7). Nanoparticles co-displaying antigen and Siglec-8 ligands inhibit antigen-specific, IgE-mediated MC activation *in vitro* and suppress anaphylaxis in siglec-8 transgenic murine models (8). Though this particular study by Duan et al. highlights inhibition of MCs sensitized to the food allergen, chicken egg ovalbumin (OVA) (8), no studies have explored whether targeting Siglec-8 or CD300a impacts *in vitro* MC activation in response to peanut.

This may be due, in part, to the paucity of standardized, *in vitro* assays that consistently demonstrate the ability of a clinically relevant food allergen to induce MC degranulation. Current *in vitro* models of the allergy effector phase use purified human IgE antibodies whose antigen specificities are unknown (9), or human plasma or serum from allergic subjects to sensitize MCs in culture (10, 11). Anti-human IgE antibodies used to crosslink IgE-FcεRI complexes on the MC can induce degranulation. However, MCs sensitized with human sera, containing anti-food allergen IgE, do not always degranulate when a specific food allergen is used as the crosslinking stimulus (9, 12).

Additionally, with 30% of patients having more than one food allergy (13), the use of human plasma to sensitize MCs creates concern for reproducibility due to variation in IgE levels and IgE specificity to multiple allergens (12). Worth noting, IgE affinity, concentration, and clonality also influence the ability of sensitized cells to degranulate (14). Lastly, studies have identified non-responder populations amongst primary mast cells and basophils isolated from allergic patients incapable of degranulating in *in vitro* assays upon antigen stimulation (15).

Outside of IgE, other immunoglobulins are also present in human plasma, including IgG antibodies, which account for 70% of antibodies in human plasma (16). IgG can downregulate MC activation by binding to inhibitory receptors, like FcγRIIB (17). This, in turn, may blunt the activation of MCs in *in vitro* assays, impacting the reproducibility of allergen-induced MC activation. This may also enhance or override potential inhibition when evaluating engagement of inhibitory receptors, like CD300a or Siglec-8. Thus, alternative *in vitro* models of food-allergen induced MC degranulation are needed to study inhibitory receptors effectively and assess the direct effects of potential therapeutics that target MC inhibitory receptors.

To address this issue, we have designed a unique *in vitro* system to mimic the effector phase of peanut allergy using novel, naturally occurring, peanut-specific human IgE monoclonal antibodies (mAbs) created *via* human hybridoma techniques (18) for sensitization of an established effector cell line. Unlike mAbs created *via* recombinant technologies, hybridoma produced mAbs maintain their heavy and light chain pairings as well as their post-translational modifications (19, 20). This is ideal for potent FcϵRI binding and cell activation (21). In our study, we assess functionality of two novel human IgE mAbs, specific for peanut antigen components including, Ara h 2, one of the most allergenic protein components of peanut (22, 23). We use this system to show that novel peanut-specific human IgE mAbs allow for direct, reproducible effector cell sensitization, activation, and degranulation in response to peanut. We also demonstrate how this model can be used as a screening tool for potential therapeutics that can blunt peanut-specific effector cell activation.

## Results

### Allergen specificity of human IgE clones generated by hybridoma techniques

Allergen specificity of human IgE clones 2C9, 16A8, 2F10 and 4C8 were determined using solid phase ImmunoCAP assay to measure binding of IgE mAbs to peanut and peanut components (Ara h 1, 2, 3, 6, 8, and 9), dust mite, and Der p 2 (**Table E1**). IgE mAb 16A8 bound to peanut (1423 kUA/L) and peanut components Ara h 2 (1440 kUA/L) and Ara h 6 (1897 kUA/L) with the highest affinity. IgE mAb 2C9 bound to peanut (549 kUA/L) and Ara h 6 (793 kUA/L) with the highest affinity. Similarly, IgE mAbs 2F10 and 4C8 bound to dust mite (2951 kUA/L and 3103 kUA/L, respectively) and Der p 2 (2756 kUA/L and 3937 kUA/L respectively) with highest affinity.

### Human peanut IgE mAbs bind to IgE receptors on RBL SX-38 cells

**Figure 1:**
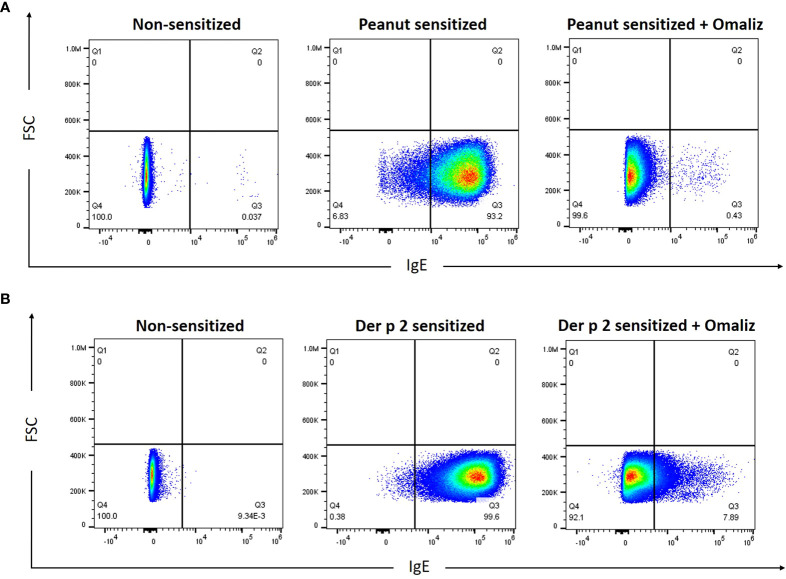
Peanut-specific human IgE mAbs bind to RBL SX-38 cells. RBL SX-38 cells were sensitized overnight with **(A)**, Peanut IgE mAbs (500 ng/mL each of clones 16A8 and 2C9) or **(B)**, Der p 2 IgE mAbs (500ng/mL each of clones 2F10 and 4C8) with or without 250 nM omalizumab (“Omaliz”). Frequency of IgE^+^ RBL SX-38 cells was evaluated *via* flow cytometry.

Rat basophilic leukemia (RBL) SX-38 cells (24), commonly used as an allergy effector cell model, express the human IgE receptor, FcϵRI (12). To examine the ability of peanut IgE mAbs 16A8 and 2C9 to bind to the human FcϵRI, RBL SX-38 cells were incubated overnight with both IgE clones in the presence or absence of omalizumab, a humanized IgG1 (5% murine, 95% human) anti-IgE mAb that binds the third constant domain of IgE and prevents IgE from binding FcεRI (25). Using flow cytometry, we found that the frequency of IgE^+^ RBL SX-38 cells was 93.2% when cells were sensitized simultaneously with both peanut IgE mAbs (**Figure 1A**). When omalizumab was added during the sensitization process, the frequency of IgE^+^ RBL SX-38 cells seen on flow cytometry fell to 0.43%, demonstrating that these human IgE mAbs bind to the surface of RBL SX-38 cells *via* FcεRI. Other RBL SX-38 cells were sensitized with 2F10 and 4C8, human IgE mAbs against Der p 2, the major allergenic component of the inhaled aeroallergen dust mite (*Dermatophagoides pteronyssinus)* (26) and examined for IgE on the cell surface in the presence or absence of omalizumab. The frequency of IgE^+^ RBL SX-38 cells was 99.6% when cells were sensitized simultaneously with both Der p 2 IgE clones 2F10 and 4C8 (**Figure 1B**). The frequency of IgE^+^ RBL SX-38 cells seen on flow cytometry fell to 7.89% when cells were sensitized with both Der p 2 IgE mAbs in presence of omalizumab. Binding of IgE clones 2C9 and 16A8 to the human FcϵRI on RBL SX-38 cells was blocked to a greater extent than the binding of IgE clones 2F10 and 4C8 when omalizumab was added during the sensitization phase (**Figures 1A, B**). This was not due to differences in allergen specificity of the IgE clones. The binding of peanut specific IgE clone 38B7, generated from a separate peanut-allergic patient, to the human FcϵRI dropped from 99% to 10.9% (data not shown). This drop was similar in magnitude to the drop seen in dust mite specific IgE clones 2F10 and 4C8 binding to human FcϵRI in the presence of omalizumab (**Figure 1A**).

### Peanut stimulation triggers allergen-specific degranulation, increased gene expression, and cytokine production in RBL SX-38 cells sensitized with peanut IgE monoclonal antibodies

**Figure 2:**
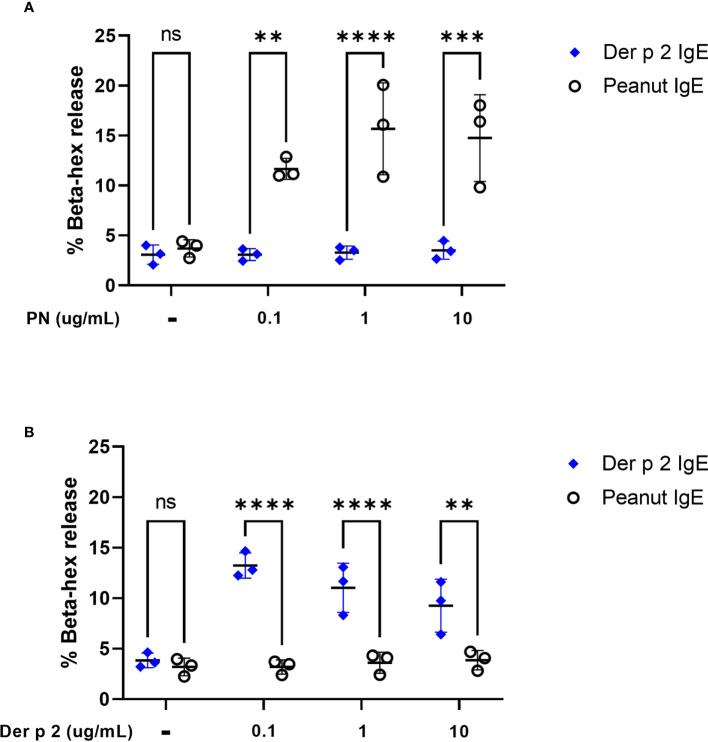
Peanut stimulation induces degranulation in peanut IgE sensitized RBL SX-38 cells. RBL SX-38 cells sensitized with peanut IgE mAbs (black) or Der p 2 IgE mAbs (blue) were stimulated with **(A)**, peanut or **(B)**, Der p 2 for 45 minutes and beta-hexosaminidase release measured. Data points reflect mean values from 3 biological replicates and are analyzed using two-way ANOVA. **p < 0.01; ***p < 0.001; ****p < 0.0001; *ns*, not significant.

To evaluate the ability of peanut IgE mAbs to induce allergen-specific effector cell degranulation, we sensitized RBL SX-38 cells overnight with peanut or Der p 2-specific IgE mAbs. Cells were stimulated with either whole peanut extract or Der p 2 antigen at varying concentrations and beta-hexosaminidase release was measured. RBL SX-38 cells sensitized with peanut IgE mAbs degranulated in a dose-dependent manner following stimulation with peanut extract (**Figure 2A**) and did not degranulate in response to Der p 2 (**Figure 2B**). In addition, cells sensitized with Der p 2 IgE mAbs, only degranulated when stimulated with Der p 2 (**Figure 2B**) and not with peanut (**Figure 2A**), establishing the allergen specificity of the peanut IgE and Der p 2 IgE mAbs, respectively.

**Figure 3:**
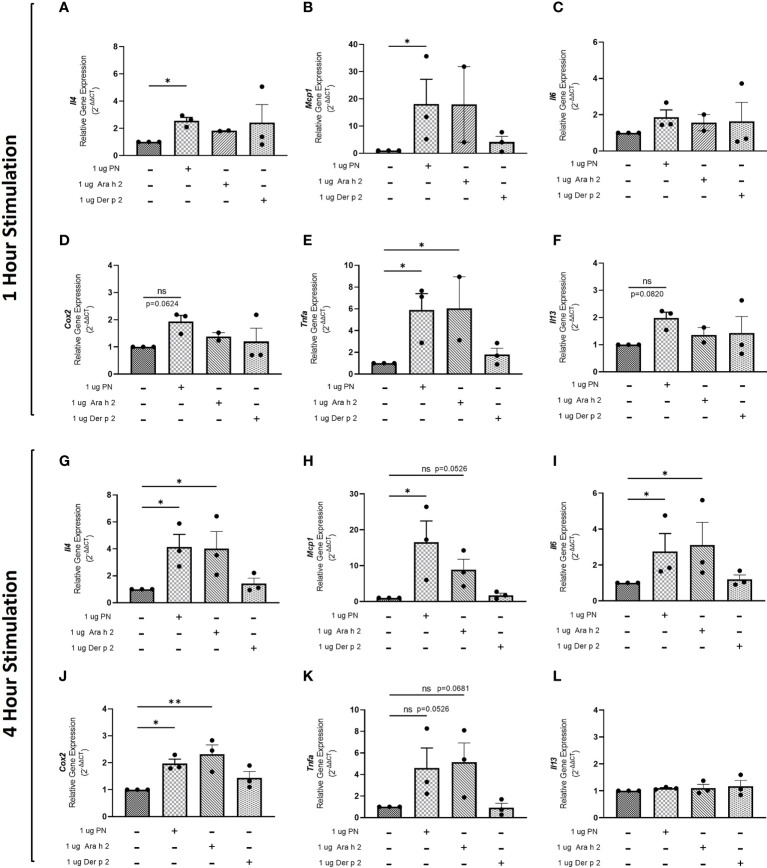
Peanut stimulation induces proinflammatory gene expression in peanut IgE-sensitized RBL SX-38 cells. RBL SX-38 cells sensitized with peanut IgE mAbs 16A8 and 2C9 were stimulated with buffer, peanut, Ara h 2, or Der p 2 for 1 hour **(A–F)** or 4 hours **(G–L)** and expression of genes encoding IL-4 **(A, G)**; MCP-1 **(B, H)**; IL-6 **(C, I)**; COX-2 **(D, J)**; TNF-alpha **(E, K)**; and IL-13 **(F, L)** assessed *via* qPCR. Kruskal-Wallis test comparing each sensitized, stimulated group to the sensitized, unstimulated control group, *p < 0.05; **p < 0.01; *ns*, not significant.

**Figure 4:**
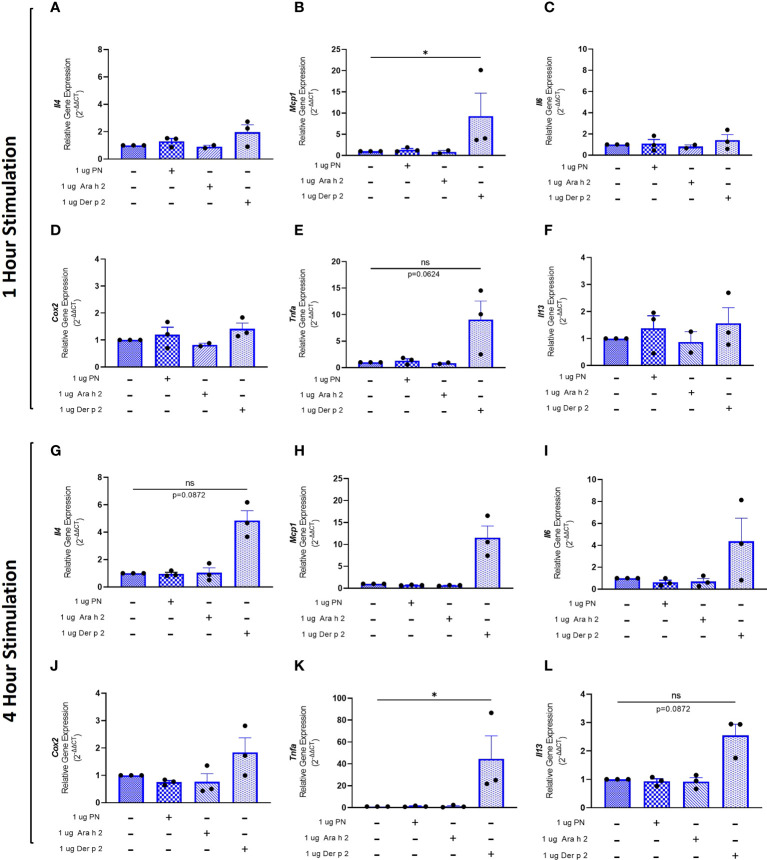
Der p 2 stimulation increases proinflammatory gene expression in Der p 2 IgE sensitized RBL SX-38 cells. RBL SX-38 cells sensitized with Der p 2 IgE mAbs 2F10 and 4C8 were stimulated with buffer, peanut, Ara h 2, or Der p 2 for 1 hour **(A–F)** or 4 hours **(G–L)** and expression of genes encoding IL-4 **(A, G)**; MCP-1 **(B, H)**; IL-6 **(C, I)**; COX-2 **(D, J)**; TNF-alpha **(E, K)**; and IL-13 **(F, L)** assessed *via* qPCR. Kruskal-Wallis test comparing each sensitized, stimulated group to the sensitized, unstimulated control group, *p < 0.05; *ns*, not significant.

To evaluate whether sensitization using peanut mAbs drives increased proinflammatory gene expression, RBL SX-38 cells were sensitized with peanut IgE mAbs (**Figure 3**) or Der p 2 IgE mAbs (**Figure 4**) and stimulated with 1 µg/mL peanut extract, Ara h 2, or Der p 2 for one hour or 4 hours. Gene expression of *Il4*, *Mcp1*, *Il6*, *Cox2*, *Tnfa*, and *Il13* was assessed *via* qPCR.

Statistically significant increases in *Il4* (**Figure 3A**), *Mcp1* (**Figure 3B**), and *Tnfa* (**Figure 3E**) gene expression were observed in RBL SX-38 cells sensitized with peanut IgE mAbs and stimulated with peanut for one hour compared to unstimulated, sensitized cells. We also observed increased expression of genes encoding the cytokine IL-13 (**Figure 3F**) and the prostaglandin endoperoxide synthase COX-2 (**Figure 3D**) after one hour of peanut stimulation, although it did not reach statistical significance. One-hour stimulation with peanut induced minimal to no change in *Il6* expression (**Figure 3C**). Notably, a statistically significant increase in *Tnfa* gene expression was also observed one hour after stimulation with Ara h 2 (**Figure 3E**).

Four-hour stimulation with peanut led to statistically significant increases in *Il4* (**Figure 3G**), *Mcp1* (**Figure 3H**), *Il6* (**Figure 3I**), and *Cox2* (**Figure 3J**) gene expression in RBL SX-38 cells sensitized with peanut IgE mAbs. Increases were also observed in *Tnfa* gene expression after 4-hour stimulation with peanut (p=0.0526) (**Figure 3K**). Additionally, 4-hour stimulation with Ara h 2 led to statistically significant increases in *Il4* (**Figure 3G**), *Il6* (**Figure 3I**), and *Cox2* (**Figure 3J**). Increases were also observed in *Mcp1* (p=0.0526) and *Tnfa* (p=0.0681) gene expression in response to Ara h 2 stimulation (**Figures 3B, K** **)**.

RBL SX-38 cells sensitized with Der p 2 IgE mAbs and stimulated with Der p 2 for one hour led to a statistically significant increase in *Mcp1* (**Figure 4B**) gene expression compared to unstimulated, sensitized cells. *Tnfa* (**Figure 4E**) gene expression also increased, although it did not reach statistical significance. Minimal to no changes were observed in *Il4* (**Figure 4A**), *Il6* (**Figure 4C**), *Cox2* (**Figure 4D**), and *Il13* (**Figure 4F**) gene expression.

Four-hour stimulation with Der p 2 led to a statistically significant increase in expression of *Tnfa* (**Figure 4K**). However, increased expression of *Il4* (**Figure 4G**), *Mcp1* (**Figure 4H**), *Il6* (**Figure 4I**), *Cox2* (**Figure 4J**), and *Il13* (**Figure 4L**) was observed, although it did not reach statistical significance.

**Figure 5:**
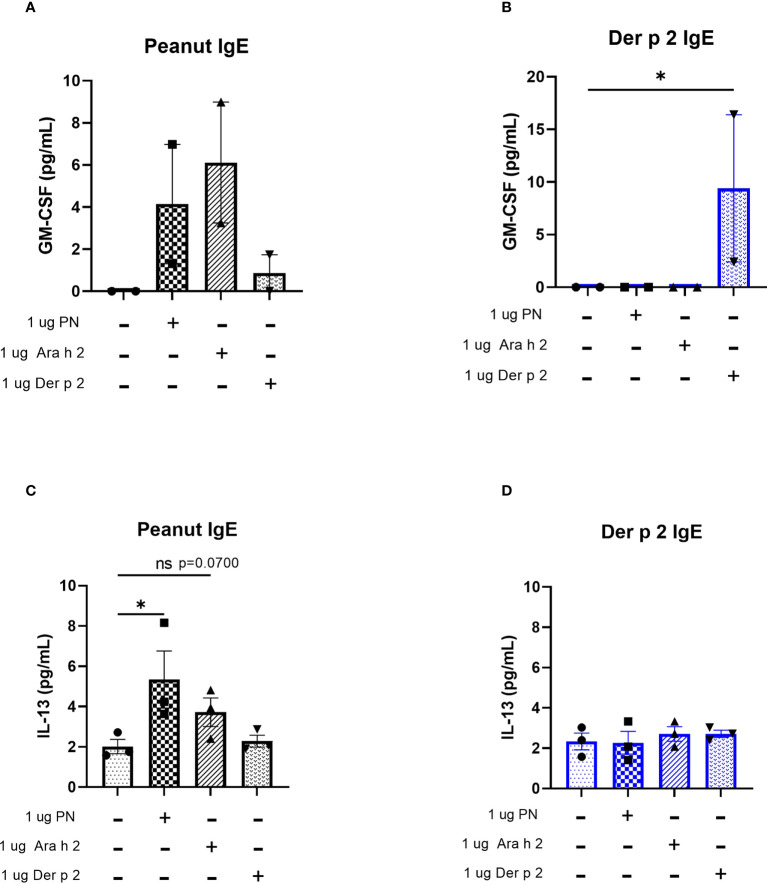
Peanut stimulation induces cytokine production in peanut IgE-sensitized RBL SX-38 cells. RBL SX-38 cells sensitized with peanut or Der p 2 IgE mAbs were stimulated with peanut or Der p 2 for 24 hours and **(A, B)**, GM-CSF production measured by ELISA and **(C, D),** IL-13 production measured *via* Luminex. **(A, B)** representative of 2 biological replicates. **(C, D),** representative of 3 biological replicates. Kruskal-Wallis test comparing each sensitized, stimulated group to the sensitized, unstimulated control group, *p < 0.05; *ns*, not significant.

Protein levels of the cytokines IL-4 and TNF-alpha were too low to be detected *via* ELISA (data not shown). However, increased amounts of GM-CSF were produced by cells sensitized with peanut IgE mAbs and stimulated with 1 µg/mL peanut or 1 µg/mL Ara h 2 antigen compared to unstimulated cells (**Figure 5A**), although these differences did not reach statistical significance. Statistically significant increases in GM-CSF were also observed for Der p 2 sensitized cells stimulated with 1 µg/mL Der p 2 antigen when compared to unstimulated cells (**Figure 5B**).

IL-13 concentrations in cell culture supernatants of RBL SX-38 cells sensitized with peanut IgE mAbs (**Figure 5C**) or Der p 2 IgE mAbs (**Figure 5D**) and stimulated with peanut, Ara h 2, or Der p 2 were assayed using a Luminex multiplex cytokine assay. Peanut stimulation led to statistically significant increases in IL-13 production by peanut-sensitized cells, compared to unstimulated, peanut-sensitized cells. Additionally, Ara h 2 stimulation led to increased IL-13 concentrations in cell culture supernatants (p=0.0700) of peanut IgE-sensitized RBL SX-38 cells. However, RBL SX-38 cells sensitized with Der p 2 IgE mAbs and stimulated with Der p 2 antigen did not induce production of IL-13 above background (**Figure 5D**).

### Peanut stimulation induces phosphorylation of Syk and Erk proteins in RBL SX-38 cells sensitized with peanut IgE mAbs

**Figure 6:**
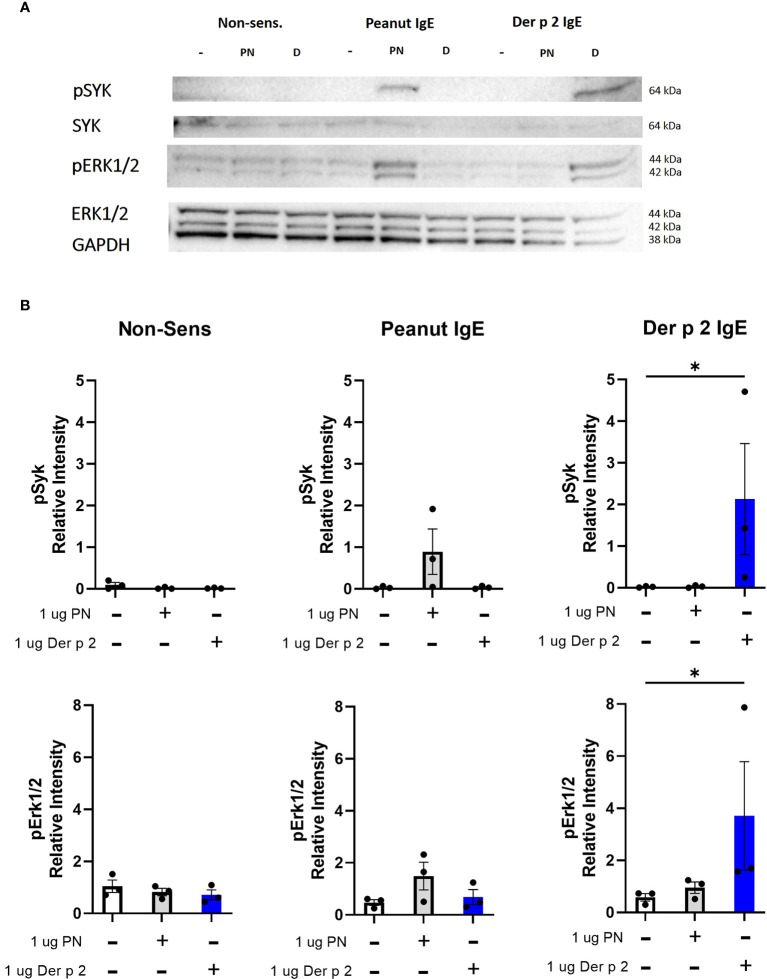
Peanut stimulation induces phosphorylation of Syk and Erk1/2. RBL SX-38 cells sensitized with either peanut- or Der p 2-specific IgE mAbs were stimulated for 5 minutes with buffer, peanut, or Der p 2 antigen before measuring changes in phosphorylation of Syk and Erk **(A)**. **(B)**, Relative intensity of bands in **(A)**. Immunoblot **(A)** is a representative of 3 biological replicates. Data included in B include all 3 biological replicates. Kruskal-Wallis test comparing each stimulated group to the matched unstimulated control group, *p < 0.05.

Crosslinking IgE leads to phosphorylation of signaling proteins like Syk and Erk downstream of FcϵRI (4). Stimulation with peanut enhanced phosphorylation of Syk and Erk1/2 in cells sensitized with peanut IgE mAbs compared to sensitized, unstimulated cells, although it did not reach statistical significance (**Figures 6A, B**). Stimulation with Der p 2 led to significant increases in phosphorylation of Syk and Erk1/2 in cells sensitized with Der p 2 IgE mAbs compared to sensitized, unstimulated cells. Enhanced phosphorylation of Syk or Erk was not observed in non-sensitized cells stimulated with peanut or Der p 2 antigen (**Figures 6A, B**).

Taken together, these data support the functionality of 16A8 and 2C9 human peanut IgE monoclonal antibodies. These IgE clones reproducibly and specifically sensitize RBL SX-38 cells to peanut and promote activation and degranulation following peanut stimulation. Thus, we turned our focus towards select cell surface inhibitory receptors, to demonstrate the utility of this system in exploring potential therapeutics for food allergy.

### Engaging inhibitory receptors CD300a and Siglec-8 reduces IgE-mediated degranulation in RBL SX-38 cells sensitized with peanut IgE mAbs

We used our model of the effector phase of peanut allergy to assess the impact of engaging inhibitory receptors CD300a and Siglec-8 on peanut-induced effector cell activation. *Cd300a* gene expression has been previously reported in rat mast cells (27). The *Siglec8* gene is conserved in rat and orthologous to human *SIGLEC8*, with low-level expression in multiple tissues, including gastrointestinal tract, respiratory, circulatory, and hemolymphoid systems (28). We have also verified detection of CD300A and SIGLEC-8 protein in the RBL SX-38 cells (data not shown).

**Figure 7:**
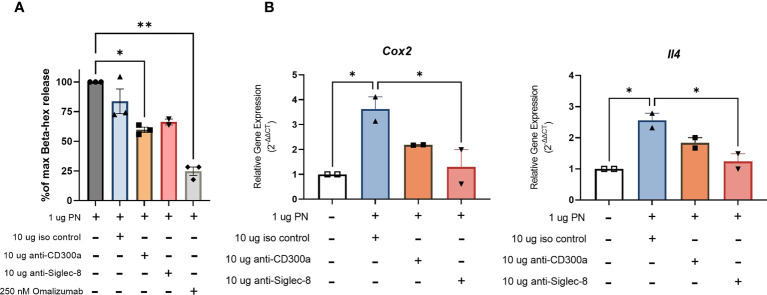
Engaging CD300a and Siglec-8 receptors reduce RBL SX-38 cell activation in peanut IgE-sensitized effector cells. RBL SX-38 cells sensitized with peanut-specific IgE mAbs 16A8 and 2C9 and stimulated with peanut were immediately treated with anti-CD300a, anti-Siglec-8, or isotype control antibodies. **(A)**, beta-hex release. **(B)**, Expression of genes encoding COX-2 and IL-4. Omalizumab added during sensitization served as a positive control for inhibition. Data shown are representative of at least 2 biological replicates. Kruskal-Wallis test comparing each group to the sensitized, peanut stimulated positive control in **(A)** and comparing each group to the isotype control treated group in **(B)**, *p < 0.05; **p < 0.01.

RBL SX-38 cells sensitized with peanut IgE mAbs were stimulated with peanut and treated simultaneously with either 10 µg/mL anti-CD300a, anti-Siglec-8, or an isotype control. After 45 minutes of stimulation/treatment, beta-hexosaminidase release was measured. Cells treated with anti-CD300a demonstrated significantly decreased degranulation compared to untreated cells. Cells treated with anti-Siglec-8 also released less beta-hexosaminidase although this difference did not reach statistical significance (**Figure 7A**). Degranulation of RBL SX-38 cells treated with 250 nM omalizumab during sensitization was used as a positive control for inhibition and showed minimal degranulation (**Figure 7A**). Treating peanut-stimulated RBL SX-38 cells with anti-Siglec-8 antibodies also led to significantly decreased expression of the genes encoding IL-4 and COX-2 (**Figure 7B**) when compared with isotype control treated cells. Treating peanut-stimulated RBL SX-38 cells with anti-CD300a antibodies led to decreased expression of the genes encoding IL-4 and COX-2 (**Figure 7B**) when compared with isotype control treated cells, although it did not reach statistical significance. RBL SX-38 cells sensitized with peanut IgE mAbs, stimulated with 1 µg/mL peanut and immediately treated with either anti-CD300a or anti-Siglec-8 for 45 minutes in doses ranging from 0.1 µg/mL to 10 µg/mL confirmed that the greatest inhibition was observed with the 10 µg/mL dose in this system (data not shown).

## Discussion

There is currently no cure for food allergy. There is one US Food and Drug Administration approved oral immunotherapy to treat peanut allergy, and several experimental therapies including epicutaneous and sublingual allergy immunotherapy to treat peanut and other food allergies. However, the durability of limited remission of food allergy after immunotherapy is not fully defined. Moreover, this treatment modality carries a risk of allergic reaction (4). As a result, there is still a need to develop novel therapies to treat peanut and other food allergies. In our study, we assess the functionality of two novel human peanut-specific IgE mAbs and their utility in an *in vitro* system to screen compounds that can inhibit the effector phase in peanut allergy.

To our knowledge, this study is the first to demonstrate functionality of peanut-specific human IgE mAbs generated from a B cell hybridoma immortalized from the blood of a peanut-allergic person. Earlier studies used recombinant DNA technology to create human/mouse chimeric IgE antibodies (26, 29). In these IgE chimeras, the Fc portion was derived from human IgE isotype constant domains, and the heavy-chain and light chain variable domains were cloned from the mouse hybridomas 2B12 and αDpX that produced murine IgE specific for Der p 2 (26). These recombinant Der p 2-specific mouse/human chimeric IgE clones were subsequently used in an indirect basophil activation test to describe the relevance of IgE properties to allergic effector cell degranulation (30).

By contrast, the human IgE monoclonal antibodies used in this study are fully human and not derived *via* recombinant DNA technology. They have naturally occurring heavy and light chain pairings dictated by genes in the nucleus of an IgE-encoding memory B cell identified from the peripheral blood of a peanut-allergic human. The peanut epitopes recognized by these IgE mAbs are clinically-relevant and immunogenic since the peanut specific IgE mAbs are clones of IgE generated in a peanut-allergic human mounting an allergic response to peanut (18). All post-translational modifications, including glycosylation which is critical for adequate degranulation (21), mirror that described for endogenous human IgE (18). We show that these antibodies bind to human FcεRI, sensitizing allergic effector cells *in vitro.* Importantly, these peanut-specific human IgE mAbs can be crosslinked directly with the clinically relevant food allergen peanut, reproducibly inducing allergic effector cell activation and degranulation. Deploying these novel allergen-specific human IgE mAbs and clinically relevant allergens in this widely-used, RBL SX-38 *in vitro* allergic effector model has the potential to expand the number of standardized tools available to sensitize and to activate sensitized allergic effector cells *in vitro* in a reproducible, allergen-specific fashion.

Human IgE mAbs generated using human B cell hybridoma techniques specific for environmental aeroallergens, including aspergillus, have been used to demonstrate the variability of allergen concentrations in commercialized allergen extracts used for immediate hypersensitivity skin testing and subcutaneous immunotherapy (18). Chicken egg ovalbumin (OVA)-specific human IgE mAb created using the same techniques have been used in passive systemic anaphylaxis models in transgenic mice expressing the human FcεRI (8), showing the utility of these human IgE mAbs in driving anaphylaxis *in vivo*. Our study expands and complements these previous reports by introducing an additional application for a human IgE mAb specific for the widely studied food allergen peanut – the creation of an *in vitro* screening platform to identify compounds with the ability to blunt allergic effector cell degranulation in the effector phase of food allergy.

Immunoreceptor tyrosine-based inhibition motif (ITIM)-containing inhibitory mast cell surface receptors have been proposed as possible pharmaceutical targets to block mast cell activation and degranulation in food allergy (4). We observed that using monoclonal antibodies specific for two ITIM-containing receptors, CD300a and Siglec-8, on allergic effector cells sensitized with peanut-specific IgE, blunted allergen-induced degranulation of these cells. Our results with Siglec-8 support the proof of concept of our *in vitro* system to identify inhibitors of mast cell degranulation. They mirror previously published reports highlighting the inhibitory effects of Siglec-8 engagement on RBL and mast cell degranulation *in vitro* and OVA-specific passive systemic anaphylaxis *in vivo* (8). Our data also suggest that targeting the CD300 receptor family on allergic effector cells, specifically the inhibitory receptor CD300a, is a potential therapeutic strategy for inhibiting peanut-specific allergic effector cell activation and degranulation. This data additionally supports previously published work showing the inhibitory effects of CD300a engagement in allergic effector cells *in vitro* (5, 31, 32) and in allergic *in vivo* models (5).

ITIM-containing inhibitory receptors act through ITIMs by recruiting phosphatases to dephosphorylate signaling proteins downstream of the FcϵRI (4). The mechanism behind Siglec-8-mediated inhibition of mast cells has been previously reported (33) and involves direct interactions between Siglec-8 and signaling molecules downstream of FcϵRI, and interactions of its ITIM with the phosphatase SHP-2 after Siglec-8 is phosphorylated (33). Recruitment of SHP-1 and SHIP has also been previously reported as the mechanism for CD300a mediated inhibition in mast cells (32). Future studies using our model system will determine whether those phosphatases are associated with CD300a and Siglec-8 signaling in the RBL SX-38 effector cell line. Whether RBL cell lines function as adequate models of rodent or human mast cells is controversial (34, 35). Thus, future studies will also deploy allergen-specific human IgE mAbs to sensitize human mast cell lines and primary human mast cell cultures. This may help to determine the relevance in human cells of targets for effector cell inhibition identified using rodent effector cells.

Our system allows for observation of the direct effects of potential therapeutics on effector cell activation by removing variables introduced when human plasma is used for sensitization. Because it excludes non-relevant IgE, multiclonal allergen-specific IgE, and other antibody subclasses, our platform simplifies the allergic effector phase in food allergy and facilitates the rapid screening of candidates for effector cell inhibition in food allergy. By no means does our model obviate the role for more complex *in vitro* and *in vivo* systems for subsequent evaluation of candidate inhibitors, since the influence of polyclonal IgE, other antibody isotypes, and non-immunoglobulin components like complement should be taken into account when developing a mast cell inhibitor applicable to allergic human patients. Our method deploys novel peanut-specific human IgE monoclonal antibodies to enable reproducible *in vitro* models of the effector phase in food allergy as a first step in the effort to unveil new therapeutic targets to prevent mast cell activation in IgE-mediated allergy. Additional studies are required to determine the clinical relevance and the mechanisms driving the inhibition observed; however, our findings represent a key starting point for identifying potential therapeutic targets on MCs and basophils and understanding how they may blunt allergen-induced effector cell activation.

## Materials and methods

### Cell culture

We received permission to use the RBL SX-38 cells from Dr. Jean-Pierre Kinet (Beth Israel Deaconess Hospital) and the cells were shared with us by Dr. Michelle Hernandez (Chapel Hill). RBL SX-38 cells were maintained at 37° C in 5% CO_2_. Cells were cultured in 1X MEM (Thermofisher, Waltham, MA), supplemented with 1.2 mg/mL geneticin (Gibco-Thermofisher), 2mM L-glutamine, 100 U/mL penicillin and 100 ug/mL streptomycin (Gibco-Thermofisher), and 10% heat-inactivated Cytiva Hyclone FBS (Fisher Scientific, Pittsburgh, PA). During overnight sensitization steps for all experiments, complete media was replaced with media without geneticin and Cytiva FBS as previously described (36).

### Human IgE monoclonal antibodies

Peanut IgE mAbs (16A8, 2C9, 38B7) and Der p 2 IgE mAbs (2F10 and 4C8) were developed and gifted by Scott A. Smith using human hybridoma techniques as previously described (18) and are available from the author by request.

### Flow cytometry

Approximately 1 x 10^6^ RBL SX-38 cells/condition were sensitized overnight with peanut IgE mAbs (500 ng/mL each) or Der p 2 IgE mAbs (500 ng/mL each) in the presence or absence of 250 nM omalizumab (Genentech, San Francisco, CA and Novartis, Basel, Switzerland). Cells were then washed and stained with eBioscience Fixable Viability Dye eFluor 450 (Thermofisher) and human IgE clone MHE-18-Alexa Fluor 647 (BioLegend, San Diego, CA). Cells were washed again, fixed, and acquired on an Attune NxT acoustic focusing cytometer (Life Technologies-Thermofisher). Flow cytometry data were analyzed using FlowJo software (Becton, Dickinson & Company, Ashland, OR).

### Allergens

*Peanut Extract-* Peanut extract was prepared by extracting peanut protein from roasted defatted peanut flour (Golden Peanut, Alpharetta, GA) as previously described (37).

*Ara h 2-* Ara h 2 allergen was gifted and prepared in the lab of Dr. Soheila Maleki as previously described (38, 39).

*Der p 2-* Natural Der p 2 (NA-DP2-1) allergen was purchased from Indoor Biotechnologies (Charlottesville, VA).

### Western blotting

2 x 10^6^ RBL SX-38 cells were sensitized with two peanut IgE mAb clones (16A8 and 2C9, 500 ng/mL each) or two Der p 2 IgE mAb clones (2F10 and 4C8, 500 ng/mL each). Cells were then washed with NaPIPES buffer (25 mM disodium PIPES, 100 mM NaCl, 5 mM KCl, 0.4 MgCl_2_, 1 mM CaCl_2_, 5.6 mM glucose, 1% BSA, pH 7) before stimulating with 1 µg/mL whole peanut extract, Der p 2, or buffer for 5 minutes. Stimulated cells were lysed on ice for 30 minutes. A bicinchonic acid (BCA) protein assay was performed on cell lysates to determine total protein content. Equal amounts of total protein were loaded onto SDS-PAGE gels and transferred to polyvinylidene difluoride (PVDF) membranes. Membranes were blocked in tris buffered saline with tween (TBST) with 2% bovine serum albumin (BSA) for 1 hour before incubating overnight with primary antibodies: 1:1000 pSYK Antibody #2711 (Cell Signaling Technology, Danvers, MA), 1:1000 pERK1/ERK2 (Thermofisher, Catalog # 44-680G), 1:1000 total SYK (Thermofisher, Catalog # PA5-96063), or 1:500 total ERK1/2 (Thermofisher, Catalog # 44-680G). Membranes were washed with TBST, then incubated for 1 hour with secondary antibodies: 1:1000 Goat Anti-Rabbit IgG (Thermofisher, Catalog # 65-6120) or 1:1000 Goat Anti-Mouse IgG1-HRP (Southern Biotech, Birmingham, AL). Chemiluminescence was used for detection of blots *via* a Bio-Rad Chemidoc (Bio-Rad Laboratories, Hercules, CA).

### Beta-hexosaminidase Assay

A colorimetric assay to detect beta-hexosaminidase (beta-hex) release was used as previously described (12) with modifications to measure degranulation of RBL SX-38 cells. Briefly, 96 well flat bottom plates were seeded overnight with 100 µl of RBL SX-38 cells (1.2 x 10^6^ cells/mL). Cells were incubated overnight with 1 µg/mL Peanut IgE mAbs (16A8 and 2C9, 500 ng/mL of each clone) or Der p 2 IgE mAbs (2F10 and 4C8, 500 ng/mL of each clone), followed by a 45-minute stimulation with different stimuli. Supernatants and cell lysates were then incubated with a substrate for 1 hour, followed by 0.2 M glycine buffer immediately before colorimetric detection. Optical density (OD) values were determined at 450 nm and percentage beta-hex release was calculated as a measure of degranulation. For inhibition studies, sensitized cells were stimulated and immediately treated with 0.1, 1, or 10 µg/mL Rabbit IgG isotype control (Thermofisher, Catalog # 31235), 0.1, 1, or 10 µg/mL anti-CD300a (Thermofisher, Catalog # PA5-113473), or 0.1, 1, or 10 µg/mL anti-Siglec-8 (Thermofisher, Catalog # PA5-87961) for 45 minutes before measuring beta-hex release *via* supernatant and cell lysate as described above.

### ELISA and Luminex assays

24-well flat bottom plates were seeded overnight with 500 ul of RBL SX-38 cells (1.2 x 10^6^ cells/mL). Cells were incubated overnight with peanut IgE mAbs (16A8 and 2C9, 500 ng/mL of each clone) or Der p 2 IgE mAbs (2F10 and 4C8, 500 ng/mL of each clone), followed by 24-hour stimulation with buffer, peanut, Der p 2, or Ara h 2 antigen. At 24 hours, supernatants were harvested for ELISA or Luminex. GM-CSF was measured using ELISA kits from Abcam (Cambridge, UK). IL-13 was measured using Rat Luminex Discover Assay (R&D Systems, Minneapolis, MN) according to the manufacturer’s protocol.

### Quantification PCR

RBL SX-38 cells were sensitized with 1 µg/mL peanut IgE mAbs (16A8 and 2C9, 500 ng/mL of each clone) or Der p 2 IgE mAbs (2F10 and 4C8, 500 ng/mL of each clone) overnight in 12 well plates (1 x 10^6^ cells/mL). Cells were washed once with NaPIPES buffer, and then stimulated for either 1 hour or 4 hours with 1 µg/mL peanut, Ara h 2, or Der p 2 antigen. qPCR was performed as previously described in (40) and data acquired using a QuantStudio 3 (Thermofisher). Briefly, total RNA was isolated using the RNeasy Plus Kit (Qiagen, Hilden Germany). 500 ng of RNA was used for cDNA synthesis using iScript Reverse Transcription Supermix for RT-qPCR (Bio-Rad, Hercules, CA). Primers for *Il4*, *Il13*, *Il6*, *Tnfa*, *Cox2*, and *Mcp1* target genes were selected and measured using SsoAdvanced Universal SYBR Green Supermix (Bio Rad). Primer details below:

**Table:** 

***Target Gene***	***Forward Primer***	***Reverse Primer***
***Il4***	TCCACGGATGTAACGACAGC	TCATTCACGGTGCAGCTTCT
***Il13***	ATGGTATGGAGCGTGGACCT	AGCGGAAAAGTTGCTTGGAG
***Il6***	CACTTCACAAGTCGGAGGCT	TCTGACAGTGCATCATCGCT
***Tnfa***	ATGGGCTCCCTCTCATCAGT	GAAATGGCAAATCGGCTGAC
***Cox2***	TGACTTTGGCAGGCTGGATT	ACTGCACTTCTGGTACCGTG
***Mcp1***	AGCCAACTCTCACTGAAGCC	AACTGTGAACAACAGGCCCA
***Actb***	GCATTGCTGACAGGATGCAG	GTAACAGTCCGCCTAGAAGCA

### Statistical analysis

Data were analyzed using GraphPad Prism (San Diego, CA) and presented as mean ± SEM. The two-way ANOVA test was used to compare degranulation of peanut and Der p 2 sensitized cells stimulated with antigen. The Kruskal-Wallis test was used to compare different experimental groups from remaining experiments. Kruskal-Wallis analysis did not include correction for multiple comparisons. P values <0.05 were considered statistically significant.

## Data availability statement

The original contributions presented in the study are included in the article/**Supplementary Material**. Further inquiries can be directed to the corresponding author.

## Author contributions

JS contributed to the study conception and design, data collection, analysis and interpretation of results, and manuscript preparation and review. OI contributed to study design, interpretation of results, manuscript preparation and review. YZ, PY, and RG contributed to data collection. MK contributed to interpretation of results and manuscript review. SS contributed to reagent development, interpretation of results and manuscript review. AB contributed to manuscript review. All authors contributed to the article and approved the submitted version.

## Funding

This work was supported by the NIH/NIAID K08AI141691 (OI)); and R01AI155668, R01AI130459, and R21AI123307 (to SS); a 2020 American Association of Allergy, Asthma, and Immunology Foundation Faculty Development Award (OI); the National Center for Advancing Translational Sciences (NCATS), National Institutes of Health, through Grant Award Number UL1TR002489; the North Carolina Biotech Center Institutional Support Grant 2017-IDG-1025, and the National Institutes of Health 1UM2AI30836-01. MK receives funding from National Institutes of Health and Department of Defense. JS was supported by the Initiative for Maximizing Student Development (NIH grant R25GM055336). AB receives grant support to his institution from the National Institutes of Health and the Burroughs Wellcome Fund. The UNC Flow Cytometry Core Facility is supported in part by NIH P30 CA016086 Cancer Center Core Support Grant to the UNC Lineberger Comprehensive Cancer Center. The UNC Advanced Analytics Core is supported by NIH Grant P30 DK034987. Research reported in this publication was supported in part by the North Carolina Biotech Center Institutional Support Grant 2017-IDG-1025 and by the National Institutes of Health 1UM2AI30836-01. The funders had no role in the analysis, decision to prepare or to publish, the manuscript. The content is solely the responsibility of the authors and does not necessarily represent the official views of the National Institutes of Health.

## Acknowledgments

We thank the UNC Flow Cytometry Core. We thank Dr. J.P. Kinet for approval to use the RBL SX-38 cells and Dr. Michelle Hernandez for providing us with the RBL SX-38 cells. We also thank Dr. Soheila Maleki for purified Ara h 2, and Johnny Castillo Cabrera and Dr. Albert Baldwin for technical assistance with western blot imaging. We also thank Carlton Anderson of the UNC Advanced Analytics Core. Lastly, we thank Drs. Eveline Wu and Adam Leitzan for critical review of this manuscript.

## Conflict of interest

SS is an inventor on a patent (US 10,908,168) describing a method to make IgE monoclonal antibodies and receives royalties for intellectual property licenses with Indoor Biotechnologies. OI is a consultant for Blueprint Medicines and Novartis. MK receives consulting fees from Ukko Inc. AB receives royalties from UpToDate; consulting honorariums from Astella Pharma Global Development, Allergy Therapeutics (UK) Ltd, DBV Technologies, kaléo, N-Fold, LLC, ALK-Abelló, Inc and UKKO, Inc. as well as Aimmune Therapeutics, Consortia TX, Inc., and Prota Therapeutics for his service on their respective Scientific Advisory Boards. AB owns stock in Allertein and Mastcell Pharmaceuticals.

The remaining authors declare that the research was conducted in the absence of any commercial or financial relationships that could be construed as a potential conflict of interest.

## Publisher’s note

All claims expressed in this article are solely those of the authors and do not necessarily represent those of their affiliated organizations, or those of the publisher, the editors and the reviewers. Any product that may be evaluated in this article, or claim that may be made by its manufacturer, is not guaranteed or endorsed by the publisher.
