# Supplementary material for: Novel peanut-specific human IgE monoclonal antibodies enable screens for inhibitors of the effector phase in food allergy
Source: Front Immunol. 2022 Sep 29;13:974374. doi: 10.3389/fimmu.2022.974374 (PMC9556733; doi:10.3389/fimmu.2022.974374)
Supplement: Supplementary file 1 [file Table_1.docx]

Supplementary Material

# Supplementary Table

|  | **Human Monoclonal IgE Antibodies** | | | |
| --- | --- | --- | --- | --- |
|  | **Peanut** | | **Dust Mite** | |
| **ImmunoCAP** | **16A8** | **2C9** | **2F10** | **4C8** |
| **Total IgE (kU/L)** | 2857 | 1858 | 2865 | 4205 |
| **Dust Mite (kUA/L)** | 21.7 | 72.7 | 2951 | 3103 |
| **Der p 2 (kUA/L)** | 2.10 | 8.99 | 2756 | 3937 |
| **Peanut (kUA/L)** | 1423 | 549 | 143 | 14.4 |
| **Ara h 1 (kUA/L)** | 11.4 | 37.7 |  | |
| **Ara h 2 (kUA/L)** | 1440 | 11.5 |  | |
| **Ara h 3 (kUA/L)** | 47.5 | 103 |  | |
| **Ara h 6 (kUA/L)** | 1897 | 793 |  | |
| **Ara h 8 (kUA/L)** | 6.04 | 34.0 |  | |
| **Ara h 9 (kUA/L)** | 1.82 | 7.73 |  | |

**Supplemental Table E1. Allergen specificity of peanut and Der p 2 mAbs.** Peanut (16A8 and 2C9) and Der p 2 (4C8 and 2F10) monoclonal IgE specificity/reactivity to peanut, peanut components (Ara h 1, 2, 3, 6, 8, and 9), Der p 2, and dust mite was assessed via ImmunoCAP.

# Supplementary Methods

**ImmunoCAP**

Binding reactivity of peanut (16A8 and 2C9) and Der p 2 (2F10 and 4C8) IgE monoclonal antibodies to total IgE, peanut, Ara h 1, Ara h 2, Ara h 3, Ara h 6, Ara h 8, Ara h 9, dust mite and Der p 2 protein was quantified using the ImmunoCAP 250 instrument (Phadia, Uppsala, Sweden) according to manufacturer’s instructions.
